# Conditions Promoting Mycorrhizal Parasitism Are of Minor Importance for Competitive Interactions in Two Differentially Mycotrophic Species

**DOI:** 10.3389/fpls.2016.01465

**Published:** 2016-09-27

**Authors:** Martina Friede, Stephan Unger, Christine Hellmann, Wolfram Beyschlag

**Affiliations:** ^1^Department of Experimental and Systems Ecology, University of BielefeldBielefeld, Germany; ^2^Ecosystem Physiology, University of FreiburgFreiburg, Germany

**Keywords:** arbuscular mycorrhizal fungi, mutualism, parasitism, competitive interactions, mycotrophy, *Hieracium pilosella*, *Corynephorus canescens*

## Abstract

Interactions of plants with arbuscular mycorrhizal fungi (AMF) may range along a broad continuum from strong mutualism to parasitism, with mycorrhizal benefits received by the plant being determined by climatic and edaphic conditions affecting the balance between carbon costs vs. nutritional benefits. Thus, environmental conditions promoting either parasitism or mutualism can influence the mycorrhizal growth dependency (MGD) of a plant and in consequence may play an important role in plant-plant interactions. In a multifactorial field experiment we aimed at disentangling the effects of environmental and edaphic conditions, namely the availability of light, phosphorus and nitrogen, and the implications for competitive interactions between *Hieracium pilosella* and *Corynephorus canescens* for the outcome of the AMF symbiosis. Both species were planted in single, intraspecific and interspecific combinations using a target-neighbor approach with six treatments distributed along a gradient simulating conditions for the interaction between plants and AMF ranking from mutualistic to parasitic. Across all treatments we found mycorrhizal association of *H. pilosella* being consistently mutualistic, while pronounced parasitism was observed in *C. canescens*, indicating that environmental and edaphic conditions did not markedly affect the cost:benefit ratio of the mycorrhizal symbiosis in both species. Competitive interactions between both species were strongly affected by AMF, with the impact of AMF on competition being modulated by colonization. Biomass in both species was lowest when grown in interspecific competition, with colonization being increased in the less mycotrophic *C. canescens*, while decreased in the obligate mycotrophic *H. pilosella*. Although parasitism-promoting conditions negatively affected MGD in *C. canescens*, these effects were small as compared to growth decreases related to increased colonization levels in this species. Thus, the lack of plant control over mycorrhizal colonization was identified as a possible key factor for the outcome of competition, while environmental and edaphic conditions affecting the mutualism-parasitism continuum appeared to be of minor importance.

## Introduction

Interactions between arbuscular mycorrhizal fungi (AMF) and plants, namely the exchange of plant-produced carbon (C) and AMF-acquired soil nutrients (mainly phosphorus, P) are generally considered mutualistic (Smith and Read, 2008). However, research in the past 20 years has drawn a more complex picture: Johnson et al. (1997) established the theory behind a mutualism-parasitism continuum, postulating that many mycorrhizal associations may shift from beneficial to detrimental for the plant, with the outcome of the symbiosis being related to plant developmental stage and edaphic or climatic growth conditions. Support for this theory has been provided by several studies (e.g., Klironomos, 2003; Jones and Smith, 2004; Mariotte et al., 2013), leading to the general acceptance of the theory that the mutualism-parasitism continuum and the respective interactions between plant and AMF species may be of great ecological significance (Klironomos, 2003). Further, a meta-analysis by Jones and Smith (2004) showed that, at least temporarily, parasitic AMF interactions may be more common than previously thought.

Mutualism in the symbiosis is expected when nutritional benefits to the plant exceed plant C costs for fungal sustenance. In fertilized soil, however, mycorrhizal benefits may be reduced because plants can acquire sufficient nutrients in the absence of AMF, which may potentially cause increased cost:benefit ratios (Johnson et al., 1997). Similarly, reduced light leads to a lower plant C budget with investments into AMF becoming relatively more costly, while benefits remain constant, thus resulting in potential parasitism (Johnson et al., 1997). AMF parasitism will induce negative growth responses, i.e., smaller biomass production of mycorrhizal (AM) plants as compared to plants in the non-mycorrhizal (NM) state (e.g., Smith and Smith, 2013). As the ancestors of vascular plants were invariably arbuscular mycorrhizal (Wang et al., 2010), selective pressure through mycorrhizal parasitism may be among the reasons for evolutionary detachment from AMF in a variety of species (e.g., Unger et al., 2016). However, the mechanisms leading to mycorrhizal parasitism and the means to stabilize the symbiosis such as the reciprocal control of resource exchange by plants and fungi, i.e., the ability to reduce nutritional reward in non-beneficial partners (van der Heijden et al., 2015), are still a matter of debate. Smith et al. (2009) stated that mycorrhizal C costs may be insignificant to the plant if photosynthesis can meet fungal C demand. Alternatively, it has been argued that plants that are non- or negatively responsive to mycorrhiza gain less P in the mycorrhizal state, as the uptake pathway via roots may be suppressed in favor of the fungal pathway (Smith et al., 2009; Smith and Smith, 2011). Despite the actual P supply by AMF, this can still be regarded as parasitism when the outcome of the symbiosis is measured in terms of growth response (Johnson and Graham, 2013; Smith and Smith, 2013). Non-responsive plants may nevertheless benefit from the symbiosis (Egger and Hibbett, 2004; Smith and Smith, 2011), for instance by increasing competitive strength through mycorrhization (Cavagnaro et al., 2004; Smith et al., 2009), with some species only being responsive when grown with competitors. On the other hand, unfavorable C drain through AMF parasitism may lead to a reduction of competitiveness (Mariotte et al., 2013). Further, presence and degree of competition between plants may determine whether AMF act parasitically or mutualistically (Klironomos, 2003; Reynolds et al., 2005). Since AMF are able to form common mycelial networks (e.g., Smith and Read, 2008) interconnecting different plant species, they can affect plant-plant interactions belowground. Although, the importance of AMF for mediating competition (e.g., Facelli et al., 2010) has long been recognized (e.g., Wagg et al., 2011; Hart et al., 2013; Höpfner et al., 2015), our understanding of the underlying mechanisms is still poor (Smith et al., 2010), particularly regarding the role of environmental factors altering the mutualism-parasitism continuum in AMF symbioses.

An important factor to consider is mycotrophy: the dependence of the plant on AMF for nutrition (Janos, 2007). Highly mycotrophic plants may, despite their dependence on AMF, suffer more from conditions promoting parasitism than less mycotrophic plants because of their lacking ability to control root colonization in the absence of mycorrhizal benefits (Johnson et al., 1997; Smith and Smith, 2011). In contrast, less mycotrophic species (e.g., grasses) have been reported to reduce AMF root colonization under conditions promoting parasitism to avoid adverse C allocation to AMF (Grman, 2012). However, the relation between degree of mycotrophy and growth response under conditions promoting parasitism has not been well studied yet.

The mutualism-parasitism continuum may be a regulator of competitive interactions and community structure (e.g., Klironomos, 2003; Cavagnaro et al., 2004; van der Heijden and Horton, 2009), with changing light and edaphic conditions over the course of succession potentially influencing AMF impact on competitive strength of differentially mycotrophic species and therefore regulating their occurrence. This study aimed at increasing our understanding of these complex processes by disentangling the implications of potential AMF parasitism on competition for soil nutrients of differentially mycotrophic species, which, to our knowledge, has not been done before. An experiment with different combinations of AM and NM plants with or without competition of the dry-acidic grassland species *Hieracium pilosella* L. and *Corynephorus canescens* (L.) P. Beauv. was conducted under semi-natural conditions. *H. pilosella* is a highly mycotrophic forb (Wang and Qiu, 2006) with a coarse root system (Bishop and Davy, 1994) as opposed to the grass *C. canescens* being facultatively mycotrophic (Wang and Qiu, 2006) with an extensive fine-root system (Bartelheimer et al., 2006).

Our hypothesis was that (1) by experimentally manipulating light, nitrogen (N) and P availability, a gradient of environmental and edaphic conditions is created that ranges from expected mutualism to expected parasitism. Largest mycorrhizal benefits were predicted at high N and low P availability because N increases photosynthetic capacity and thereby C supply to the AMF (Johnson, 2010) and P-deficiency makes mycorrhizal nutrition extremely valuable (e.g., Hetrick, 1991; Lambers et al., 2008; Smith and Read, 2008; Treseder, 2013; Johnson et al., 2015). Detrimental effects were presumed at high P and shade due to the high nutrient availability and low C provision (Johnson et al., 1997; Grman, 2012). For example, in a study by Olsson et al. (2010), plants were C-limited but colonization was not reduced at high P levels with the AMF acting parasitically.

In detail, we hypothesized that (2) *H. pilosella* exhibits decreased mycorrhizal benefits under conditions promoting AMF parasitism due to its high mycotrophy. Nevertheless, in this species, mycorrhizal growth dependency (MGD), as a measure of AM plant biomass in relation to NM plants, is expected to remain positive in all treatments. In contrast, we anticipated no influence of AMF on the growth of the less mycotrophic *C. canescens* irrespective of the environmental conditions. We hypothesized further that (3) *H. pilosella* cannot control colonization by the AMF, while *C. canescens* may decrease mycorrhization with increasing potential for parasitism and thus may control cost:benefit ratio. Due to unfavorable cost:benefit ratios under conditions promoting parasitism, we expected (4) *H. pilosella* to lose competitive strength in interactions with *C. canescens* in the shade and P fertilization treatments.

## Materials and methods

### Experimental design

We performed a controlled field experiment using AM and NM individuals of the dry-acidic grassland species *H. pilosella* and *C. canescens*. Seeds of each species (Blauetikett-Bornträger GmbH, Offstein, Germany; Botanical Garden of the University of Münster, Germany) were sown and started in small boxes (1105 cm^3^) with sterilized (120°C for 1.5 h) sand. Two weeks after germination the seedlings were transplanted into bigger boxes (16,400 cm^3^, 218 seedlings) filled with sterilized sand. Half of the plants were inoculated using an inoculum-sand-mixture containing the AMF *Rhizophagus irregularis* (INOQ GmbH, Schnega, Germany), the other half was treated with a microbial wash, which was extracted from the inoculum by sieving the supernatant of a water-inoculum-mixture through a 20 μm sieve (Koide and Li, 1989). After 8 weeks of growth in a climate chamber (photosynthetic photon flux density ~320 μmol m^−2^s^−1^, light/dark period 14/10 h, temperature 22°C/15°C, relative humidity 65%), the plants were hardened off outside for 5 weeks. The plants were watered regularly to keep relative soil water content at ~6% and fertilized 3 times a week with 250 ml of a modified Hoagland fertilizer solution (Hoagland and Arnon, 1950) per box with increasing concentration from a dilution of 1:8 over 1:4–1:2 [3 mmol KNO_3_, 1 mmol Ca(NO_3_)_2_, 0.5 mmol (NH_4_)_2_SO_4_, 0.5 mmol (NH_4_)_2_HPO_4_, 1 mmol MgSO_4_, 0.5 mmol KCl, 0.5 mmol FeC_6_H_5_O_7_, 0.0125 μmol H_3_BO_3_, 0.001 μmol MnSO_4_, 0.001 μmol ZnSO_4_, 0.00025 μmol CuSO_4_, 0.00025 μmol MoO_3_ per liter], to adjust the application to the increasing nutrient demand of the plants.

The field experiment was conducted on a sand pit (20 m long, 6 m wide, 1.2 m deep) filled with river sand located in a common garden area next to the University of Bielefeld, Germany (52°03′3936″N, 8°49′5211″E, 124 m a.s.l.). Climate is temperate and average annual temperature and rainfall is 8.9°C and 832 mm, respectively. The sand pit is designed to mimic the natural situation of early successional stages of mid-European inland sand ecosystems and is divided into four chambers (6 m long, 5 m wide) separated by pond liner. Each chamber contains an effective draining system. For further details see Weigelt et al. (2005). Initial nitrate, ammonium and phosphate contents of the sand in the sand pit were 0.18, 0.14, and 1.9 mg kg^−1^, respectively, corresponding to an initial substrate N:P ratio of 0.17. In order to sterilize the sand for the NM treatment, two of the chambers were steamed under foil (Vaporex 3000, 30 × 2.70 m; Seifert, Kehl, Germany) for 3 h, using a steam boiler MS 200 (Seifert, Kehl, Germany; flow: 200 kg steam per hour), which led to a temperature of ~110°C in the sand to depth of at least 0.5 m. After sterilization a second microbial wash (see above) derived from the sand of the unsteamed chambers was applied to the NM plants shortly after plantation.

We used a target-neighbor approach (Gibson et al., 1999) with one target plant and six border plants planted in a hexagon with an edge length of 7 cm. Both species were planted either as single controls (i.e., without neighbors) or with interspecific or intraspecific neighbor combinations. These plots were randomized within each chamber. To induce environmental conditions potentially affecting mycorrhizal growth responses along the mutualism-parasitism continuum we chose the following treatments: Half of the plots (two chambers) were shaded with mesh excluding ~40% sunlight. Randomization of the shade and light treatments was not possible within the chambers. One third of the plots was fertilized weekly using 30 ml of a solution of 15 mmol l^−1^ of NH_4_NO_3_ per plant (N fertilization), one third of the plots received 30 ml of a solution of 4 mmol l^−1^ of NaH_2_PO_4_ per plant (P fertilization) and one third did not receive any fertilizer. There was a diverse distribution of the specific fertilization treatments among the four chambers. All plants received additional water according to demand. We hypothesized environmental and edaphic conditions to shift mycorrhizal growth response from strong mutualism to parasitism along the six different treatments: 1 = light, N fertilization, 2 = light, no fertilization, 3a = shade, N fertilization, 3b = light, P fertilization, 4 = shade, no fertilization, 5 = shade, P fertilization. This scale from 1 to 5 is henceforth referred to as parasitism scale with increasing numbers representing increasing potential to cause mycorrhizal parasitism (parasitism potential). The combinations of the two species as AM and NM single plants, in intraspecific competition and in interspecific competition, with light and shade, N fertilization, P fertilization, and no fertilization and a replication number of 5 led to a total number of 360 plots.

Soil moisture was recorded in 10 s intervals and stored as half hourly means in all chambers by a data logger CR10X (Campbell Scientific, Logan, UT, USA), using soil moisture sensors 10 HS (Decagon Devices, Pullman, Washington, USA). Air temperature, soil temperature and relative humidity was recorded half hourly by HOBO U12 Data loggers (Onset Computer Corporation, Cape Cod, Massachusetts, USA). The sensors for soil moisture and soil temperature were placed in the soil at a depth of 10 cm. The data loggers for air temperature and relative humidity were placed 2 cm above the soil and were shielded against direct sunlight and rain. Photosynthetic active radiation was measured once a week at different times of day using a LI-COR LI-250 light meter (LI-COR, Lincoln, NE, USA) with average values of 527.6 ± 47.2 and 316.1 ± 32.9 μmol m^−2^ s^−1^ in the light and shade treatments, respectively. Shade treatments did not strongly affect climate conditions other than light. Air temperature, relative humidity and soil temperature were on average 15.6 ± 0.7°C and 15.7 ± 0.7°C, 82.8 ± 2.1% and 83.5 ± 1.8%, 14.6 ± 0.3°C and 14.3 ± 0.3°C in light and shade, respectively. Soil moisture differed slightly between light and shade treatments with average values of 0.28 ± 0.001 and 0.26 ± 0.001 m^3^ m^−3^, with these values indicating sufficient water supply in all treatments. Maximum and minimum soil moistures over the experimental period were 0.30 and 0.13 m^3^ m^−3^ in the shade and 0.33 and 0.19 m^3^ m^−3^ in the light treatments, respectively. Average rainfall during the experimental period was 3.86 ± 0.82 mm d^−1^, with cumulative rainfall being consistently higher during the months of July (147 mm) and August (122 mm) than during September (64 mm). Rainfall data was measured at the weather station Bielefeld-Deppendorf (data from Wetter.com GmbH, 2014).

### Harvest and analysis of plant material

At the end of the growing season after 14 weeks of growth on the sand pit, all plots were harvested between October 13 and October 30, 2014. We used a hexagonal template in plot size with an edge length of 32.5 cm. Alongside the edges, the sand was cut off in order to carefully excavate roots and then dig out the whole plant. Following removal of all plants, the plot was searched for detached roots, which generally were *C. canescens* roots torn off due to their fragile structure. These, in comparison very small proportions, were later added to the *C. canescens* root biomass of the respective plot. Coarse sand was washed off the harvested plants, which were subsequently transferred to the lab, where they were separated into root and shoot material. Tangled root systems were gently floated in water and separated for the individual plants. Again, in some cases a very small fraction of *C. canescens* roots that could not be attributed to any of the plants within a plot, was added in equal parts to the biomass of all *C. canescens* plants of the plot. Root and shoot material was dried at 60°C and weighed.

Total dry weights of AM and NM plants were used for calculation of the species-specific MGD, according to Grman (2012). When AM plant biomass was greater than NM plant biomass, Equation (1) was applied and when NM plant biomass was greater than AM plant biomass, Equation (2) was applied:

(1) $$\begin{array}{r} \text{MGD}=100^{*}\left(1-\bar{\text{NM}}/\text{AM}\right) \end{array}$$

(2) $$\begin{array}{r} \text{MGD}=100^{*}\left(\text{AM}/\bar{\text{NM}}-1\right) \end{array}$$

where AM is the dry weight of an individual AM plant and is the mean dry weight of the corresponding NM plants. This index ranges from −100 to +100% making it particularly suitable for this experiment because both beneficial and detrimental effects of AMF are scaled equally.

The relative neighbor effect (RNE, Equation 3) was chosen as a quantitative measure of competitive strength (e.g., Callaway et al., 2002; Bartelheimer et al., 2008). RNE is a modification of the relative competitive intensity (RCI; Wilson and Keddy, 1986), with both indices being identical in the case of competitive interactions but differing in the case of facilitative interaction.

(3) $$\begin{array}{r} \text{RNE}=\left({\text{P}}_{\text{control}}-P_{mix}\right)/x \end{array}$$

$$\begin{array}{r} \text{with x}={\text{P}}_{\text{control}}\;if\;P_{control}>P_{mix}\\ \text{x}={\text{P}}_{\text{mix}}\;if\;P_{mix}>P_{control} \end{array}$$

where P_mix_ is the performance of a competing plant and P_control_ is the average performance of the corresponding single plants. Here, total dry weight was used as performance parameter. In contrast to RCI, RNE allows an equally rated assessment of competitive and facilitative interactions as the values vary between −1 and +1, with positive and negative values indicating competitive and facilitative effects, respectively (Markham and Chanway, 1996; Callaway et al., 2002).

### Quantification of mycorrhizal root colonization

Representative subsamples of the extracted roots of both, AM and NM plants were analyzed for mycorrhizal colonization. The roots were bleached in 10% KOH at 90°C for 10 min, rinsed with deionized water and stained with an ink-acetic-acid solution (1:1:8 = ink: 10% acetic-acid: H_2_O) at 90°C for 15 min (Phillips and Hayman, 1970). The root fragments were then transferred to microscope slides and the percentage of root length colonized by AMF was estimated at x 250 magnification using a modified intersect method (McGonigle et al., 1990), scoring a minimum of 100 intersections per sample for the presence of hyphae, vesicles and arbuscules.

### Quantification of plant N and P

Root and shoot fractions of dried plant material were ground in a ball-mill (Retsch MM 301, Retsch, Haan, Germany) prior to further analysis. Two to five milligrams of ground plant material was analyzed for total elemental C and N in an elemental analyzer (EuroVector, HEKAtech, Wegberg, Germany). Plant P content was measured using high-temperature oxidation and colorimetrical quantification according to Watanabe and Olsen (1965). Dried plant material was ashed at 500°C for 4 h in a muffle furnace and, after cooling, 1–3 mg of ash was digested in 10% nitric acid. The extracts were diluted with bidestilled water and analyzed for orthophosphate concentration using flow injection analysis at 880 nm (FIA-Lab II, MLE GmbH, Dresden, Germany). Tissue P and N amounts were calculated by multiplying N- and P-concentrations with total plant dry weight. Due to their small biomass, plant P could not be analyzed in each *H. pilosella* NM plant.

### Statistical analyses

To evaluate the importance of each of the factors manipulated in the experiment for the measured response variables, Partial Least Square (PLS) regressions were applied. PLS regressions are a standard tool in multivariate statistics to model the dependence structure of one or multiple response variables on a set of predictors and are particularly suitable when the number of samples is small relative to the number of predictors. Similar to PCA, the X matrix is modeled by the product of two smaller matrices, the scores and loadings. In PLS regressions, X and Y matrices are modeled simultaneously and the loading matrices are calculated such that the X and Y residuals are small while at the same time the correlation between X and Y scores is maximized (Wold et al., 1983, 2001). Since X data were categorical, dummy variables were applied by replacing each variable with the corresponding indicator matrix (Xiong and Meullenet, 2006). X and Y data were mean centered and weighted by 1/SD. PLS regressions were performed using the kernel algorithm in R 3.2.2 (R Development Core Team, 2015) with the package “pls” (Mevik et al., 2015). Ten-fold cross validation was performed. For k-fold cross-validation, the dataset is divided into k segments and the model is run k times, with each segment being left out from model calibration in one run and used for validation in this run. This allows for significance testing of the regression coefficients, as a set of coefficients is calculated for each submodel and their variation can hence be estimated. This information can be used to identify independent variables that are important for predicting the responses (Martens' Uncertainty Test; Martens and Martens, 2000).

PLS regressions were calculated for each combination of species^*^mycorrhizal status. Predictor variables were the three levels of the competition treatment, i.e., single plants, interspecific and intraspecific competition, shade treatment, and N, P and no fertilization treatments, respectively. For the AM treatments, response variables were biomass, plant N, plant P, colonization and MGD, while the latter two obviously were not modeled in the NM treatment. Additionally, as biomass in the NM treatment in *H. pilosella* was extremely low, plant P could not be analyzed for the majority of samples and thus, this variable had to be excluded in the PLS model of this group.

A minimum of 2 components was used and the optimal number of components was determined as the first minimum in a plot of root mean square error of prediction (RMSEP) against the number of components.

Further statistical analyses were performed using Statistica 6.0 (StatSoft Inc., Tulsa, USA). Data was tested for normal distribution (Shapiro–Wilk test) and homogeneity of variances (Brown–Forsythe test). Data that did not satisfy the assumptions of normal distribution was square root transformed prior to analysis. Four-way ANOVA was performed on biomass and RNE (factors: species, mycorrhization, intraspecific or interspecific competition, and parasitism scale). When significant differences were found for main effects, Fisher's LSD *post-hoc* pair wise comparison was applied to determine individual differences between means.

## Results

### Impact of conditions promoting parasitism on plant growth

**Figure 1:**
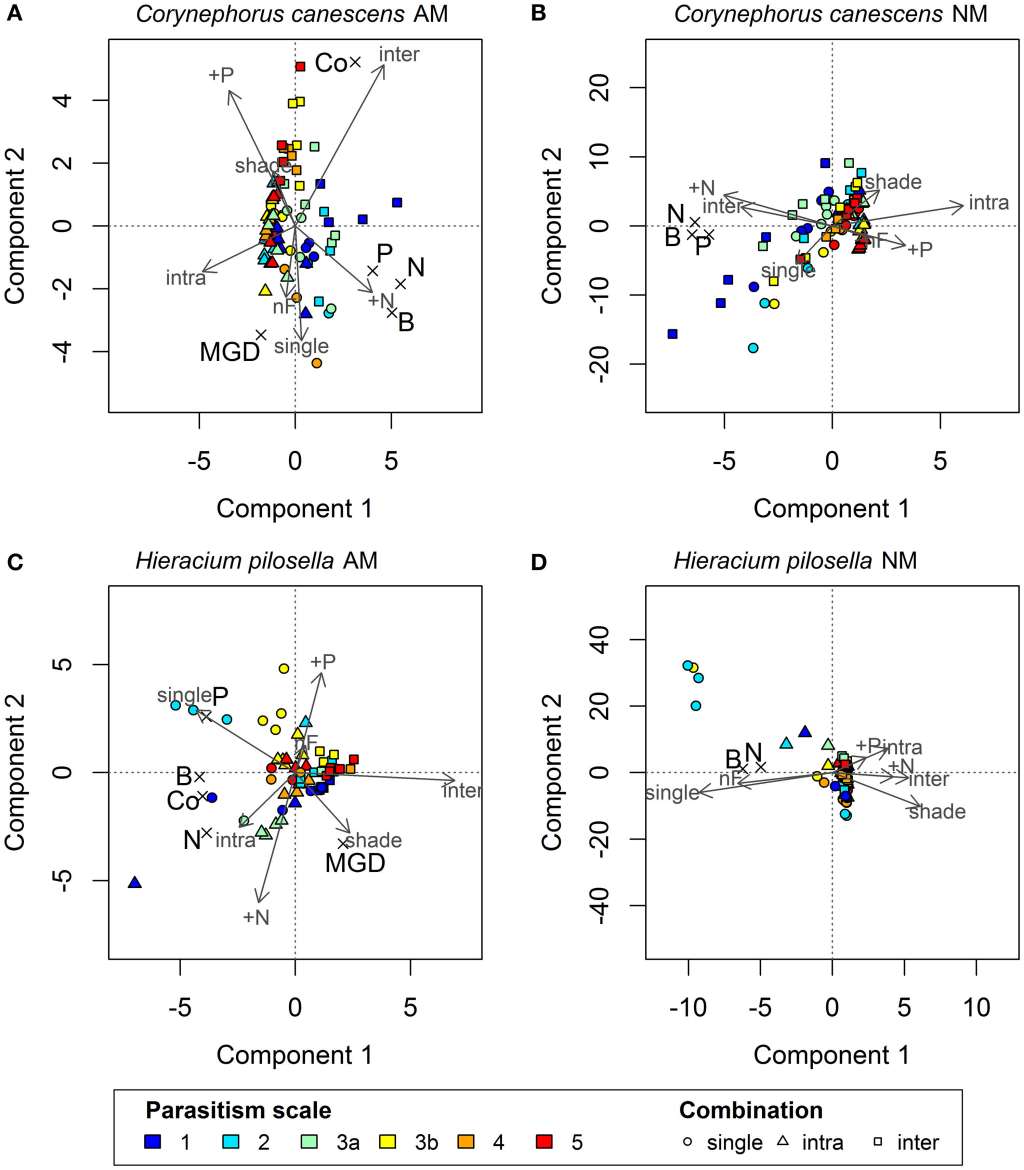
**Biplots showing Y-scores and X- and Y-loadings of the first two components of Partial Least Squares (PLS) regression models for mycorrhizal (A) and non-mycorrhizal (B) plants of ***Corynephorus canescens*** and mycorrhizal (C) and non-mycorrhizal (D) plants of ***Hieracium pilosella*****. Symbols of sample scores are colored depending on assumed ranks of a theoretical scale of mycorrhizal parasitism potential, with blue to red indicating treatments with low to high parasitism potential. Symbol types refer to competition treatments, with dots indicating plants grown alone and triangles and squares indicating intra- and interspecific competition, respectively. Scaled X-loadings are represented by gray labeled arrows. Scaled Y-loadings are depicted by crosses with black letters in larger fond. For explained variance in Y please see Table 2. AM, mycorrhizal plants; NM, non-mycorrhizal plants. Independent variables (X): inter, intra, and single = interspecific, intraspecific competition and plants grown alone; shade = shade treatment; N, P, nF = nitrogen and phosphorus fertilization and no fertilization, respectively. Dependent variables (Y): MGD = mycorrhizal growth dependency; B = total biomass; Co = colonization; P = plant phosphorus content, N = plant nitrogen content.

Increasing intensity of conditions promoting parasitism resulted in lower biomass in the AM treatment in *C. canescens*. Here, single plants produced ~4 times less biomass under P fertilization and shade (high expected parasitism) than under N fertilization and light (high expected mutualism; Supplementary Table S1). In accordance, in the biplot of the first two components of the PLS model, X-loadings of P fertilization and shade, with high parasitism potential, were located opposite to Y-loadings of biomass, which indicates that biomass was negatively related to these treatments (Figure 1A). The distribution of the sample scores of the first two components also indicated the negative relation between biomass and parasitism potential. Scores of samples in treatments with high parasitism potential (red end of color scale) had higher values on component 1 and where thus located opposite to the loadings of biomass, plant N, and plant P, while the location of samples with low parasitism potential (blue end of color scale) indicated higher values of these responses (Figure 1A). Further, P fertilization and shade treatments were identified as significant predictors for biomass, plant N, and plant P of AM *C. canescens* (Table 1). N fertilization treatment was also a significant predictor for biomass and plant N, while the factor “no fertilization” was not important for the prediction of these parameters (Table 1). However, the negative effects of parasitism promoting conditions were not related to mycorrhization, as the NM treatments of *C. canescens* showed similar dependence structure and score distribution with blue colored scores next to biomass, plant N, and plant P loadings (Figure 1B). On component 1, the loadings for shade and P fertilization were located opposite to biomass, plant N and plant P, which were explained by the first component with 48.1, 46.3, and 37.7%, respectively (Table 2). Both AM and NM *C. canescens* tended to have least biomass with P fertilization (~0.73 and ~3.50 g, respectively) and highest biomass with N fertilization (~2.13 and ~5.99 g, respectively; Supplementary Table S1), as reflected in the location of fertilization loadings as compared to biomass loadings on components 1 and 2 (Figures 1A,B). MGD in *C. canescens* single plants was markedly negative in all treatments with an average value of −59% (Supplementary Table S1), showing that AM *C. canescens* were smaller (~1.79 g) than the corresponding NM plants (~4.51 g), which was significant for AM plants with P fertilization (~0.73 g, *p* < 0.05; Supplementary Table S1). PLS indicated a negative relation between MGD and the X-loadings shade and P fertilization by the opposite position along component 2 (Figure 1A), with P fertilization being a significant predictor for MGD (Table 1).

**Table 1:** *****P***-values describing significance of the independent variables (single, intra and inter = plants grown alone, plants grown in intraspecific and interspecific competition; shade = shade treatment; N, P, nF = nitrogen and phosphorus fertilization and no fertilization, respectively) as predictors of the dependent variables (MGD, mycorrhizal growth dependency; P, phosphorus; N, nitrogen) in PLS regression models for ***Corynephorus canescens*** (CC) and ***Hieracium pilosella*** (HP) in the mycorrhizal (AM) or non-mycorrhizal (NM) state**.

**Group**	**Dependent variable**	**Single**	**Intra**	**Inter**	**Shade**	**N**	**P**	**nF**
*CC AM*	MGD	0.850	**0.013**	**0.002**	0.278	0.823	**0.007**	0.113
	Biomass	**0.014**	**0.003**	**0.045**	**0.010**	**0.001**	**0.002**	0.262
	Colonization	<**0.001**	**0.020**	**0.001**	0.974	0.673	0.333	0.595
	Plant P	0.123	**0.043**	**0.054**	**0.095**	**0.012**	**0.031**	0.314
	Plant N	0.163	**0.005**	**0.002**	**0.027**	<**0.001**	**0.002**	0.720
*HP AM*	MGD	**0.040**	0.422	**0.003**	**0.040**	**0.006**	**0.029**	0.533
	Biomass	**0.015**	0.770	**0.001**	0.226	0.358	**0.017**	0.233
	Colonization	0.339	**0.003**	<**0.001**	0.937	0.275	0.523	0.641
	Plant P	**0.010**	0.864	**0.002**	**0.033**	0.189	0.921	0.266
	Plant N	**0.086**	0.281	**0.002**	0.480	**0.005**	**0.003**	0.233
*CC NM*	Biomass	**0.016**	<**0.001**	**0.004**	**0.005**	<**0.001**	**0.024**	**0.070**
	Plant P	**0.057**	**0.001**	**0.024**	**0.006**	**0.005**	**0.044**	0.100
	Plant N	0.463	<**0.001**	**0.002**	**0.021**	<**0.001**	**0.008**	**0.009**
*HP NM*	Biomass	0.131	0.469	**0.060**	0.110	0.148	0.821	0.249
	Plant N	0.176	0.828	**0.061**	**0.063**	0.223	0.955	0.343

Significance was tested using a t-test of the regression coefficients, with their variation being estimated by jack-knifing in a cross-validation procedure (Martens' Uncertainty Test; Martens and Martens, 2000). For details please see Materials and Methods section. P ≤ 0.1 are printed bold.

**Table 2:** Cumulative Y variance explained by Partial Least Squares (PLS) regression models in % for the components used, for each dependent variable (MGD, mycorrhizal growth response; P, phosphorus; N, nitrogen) of the separate models in each combination of species^*^mycorrhizal status for ***Corynephorus canescens*** (CC) and ***Hieracium pilosella*** (HP) in the mycorrhizal (AM) or non-mycorrhizal (NM) state.

**Cumulative explained Y variance (%)**						
**Group**	**Dependent variable**	**1 Comps**	**2 Comps**	**3 Comps**	**4 Comps**	**5 Comps**
*CC AM*	MGD	4.6	21	30.6	30.7	30.7
	Biomass	35.1	45.3	45.6	48.4	48.4
	Colonization	13.6	50.2	55.9	56.1	56.1
	Plant P	23.9	26.8	27	29	29.3
	Plant N	41	45.5	45.5	45.5	47.2
*HP AM*	MGD	11.1	36.4	36.4	37	*NA*
	Biomass	28.6	28.7	33.7	34.4	*NA*
	Colonization	33.8	36	44.9	45.8	*NA*
	Plant P	26.9	37.8	38.9	39.1	*NA*
	Plant N	26	38.3	39.8	40.7	*NA*
*CC NM*	Biomass	48.1	49.9	50	*NA*	*NA*
	Plant P	37.7	39.4	40	*NA*	*NA*
	Plant N	46.3	46.7	48.1	*NA*	*NA*
*HP NM*	Biomass	22.7	23.3	*NA*	*NA*	*NA*
	Plant N	16.8	18.2	*NA*	*NA*	*NA*

AM *H. pilosella* single plants were smaller (~0.42 g) than the corresponding *C. canescens* plants (~1.79 g), which was significant for all treatments (*p* < 0.05), except for *C. canescens* with P fertilization (~0.73 g) with its relatively high parasitism potential (Supplementary Table S1). In contrast to *C. canescens*, biomass production and plant N in AM *H. pilosella* single plants was less related to potentially parasitic treatments (shade and P fertilization), with parasitism promoting conditions resulting in an only ~25% biomass decrease. This is also indicated by PLS loadings of components 1 and 2 located at a right angle to each other (Figure 1C). Furthermore, the shade treatment was no significant predictor for biomass (Table 1). As opposed to *C. canescens*, score distribution on components 1 and 2 was non-distinctive in terms of biomass. Plant P, however, was highest in light and lowest in shade (Figure 1C) with average values of 1.02 mg and 0.64 mg, respectively (Supplementary Table S1), and the shade treatment was a significant predictor for plant P (Table 1).

P fertilization was an important predictor for biomass and plant N, whereas N fertilization was significant for plant N and the factor “no fertilization” was not important for the prediction of any of the growth parameters (Table 1). However, the relation between fertilization treatments and growth parameters was not as pronounced as in *C. canescens* (Figures 1A,C).

In contrast to *C. canescens*, conditions promoting mycorrhizal parasitism resulted in no marked biomass decreases in *H. pilosella* NM plants. However, in contrast to AM plants, biomass of NM single plants tended to be higher in the light than in the shade treatments (Figure 1D, Supplementary Table S1) and the shade treatment was a significant predictor of biomass and plant N (Table 1).

Opposite to *C. canescens*, MGD in *H. pilosella* single plants was distinctly positive with an average value of 78% throughout all treatments (Supplementary Table S1). Furthermore, MGD was positively related to shading treatments (Figure 1C) with plants showing ~1.5 times higher MGD in the shade as compared to the light treatments (Supplementary Table S1). The shade treatments, as well as N and P fertilization, were significant predictors for MGD in *H. pilosella* (Table 1). As indicated by MGD values, NM *H. pilosella* single plants were significantly smaller (~0.08 g) than AM *H. pilosella* single plants (~0.42 g, *p* < 0.05). Further, in the NM state, *H. pilosella* showed significantly less biomass (~0.08 g) than *C. canescens* (~4.51 g, *p* < 0.01) in all treatments (Supplementary Table S1).

### Impact of conditions promoting parasitism on mycorrhization

In *C. canescens*, mycorrhizal root colonization was highest in the interspecific competition treatment with ~20.5% and lowest in the single treatment with only ~2.4% of root length colonized (Figure 1A, Supplementary Table S1). Moreover, competition and single treatments were significant predictors for colonization, while this was not the case for shade and fertilization treatments (Table 1). Hence, colonization was not affected by conditions promoting parasitism with an average value of 9.7% in both light and shade treatments and similar mean values in P fertilization (11%), N fertilization (9.3%), and no fertilization treatments (8.7%; Supplementary Table S1). This is also represented by score distribution on components 1 and 2 forming no clear color pattern relating to the Y-loading colonization, i.e., samples with low parasitism potential (blue end of the color scale) were not clustered with the colonization loading. Additionally, a strong negative correlation between colonization and MGD was evident, as demonstrated by loadings on the first two components being located opposed to each other (Figure 1A).

In marked contrast to *C. canescens*, colonization in *H. pilosella* was lowest in the interspecific competition treatment with ~46.3% and highest in the intraspecific competition treatment with ~70.7% (Figure 1C, Supplementary Table S1). These two treatments were the only significant predictors for colonization in *H. pilosella* (Table 1). Thus, conditions promoting parasitism influenced colonization only marginally with very similar means of 58, 61.5, 56.1, 60.2, and 62.9% in the shade, light, P fertilization, N fertilization and no fertilization treatment, respectively (Supplementary Table S1), as also indicated by the non-distinctive score distribution. In contrast to *C. canescens*, colonization was not related to MGD (Figure 1C). Neither *H. pilosella* nor *C. canescens* NM plants were colonized by AMF (Supplementary Table S1).

### Impact of conditions promoting parasitism on competition

**Figure 2:**
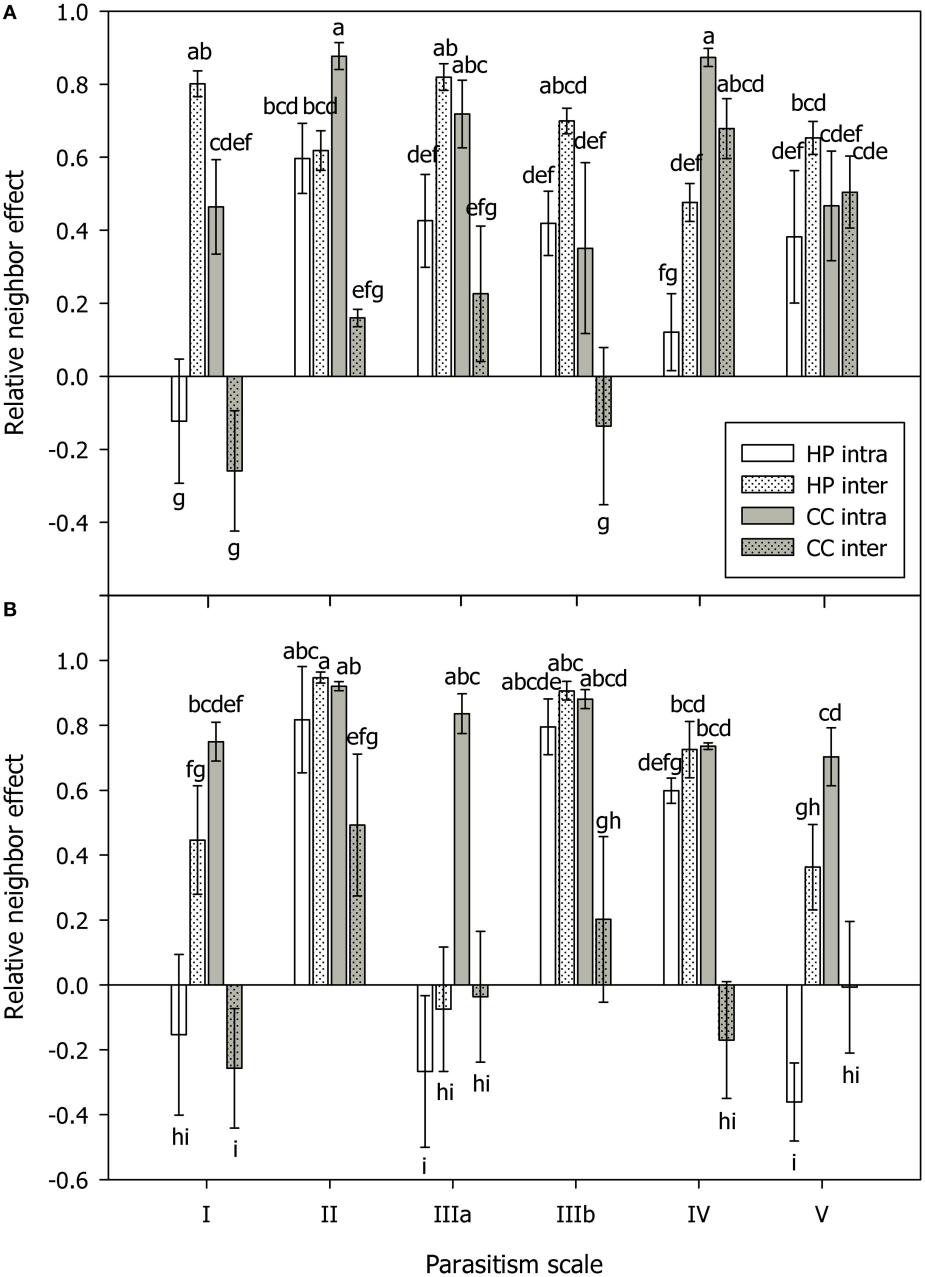
**Relative neighbor effect (RNE) in mycorrhizal (A) and non-mycorrhizal (B) plants of ***Hieracium pilosella*** (HP, white) and ***Corynephorus canescens*** (CC, gray) in intraspecific (intra, open bars) and interspecific (inter, dotted bars) competition under conditions along a theoretical scale of mycorrhizal parasitism potential (parasitism scale) with I standing for hypothesized strong mutualism to V standing for hypothesized parasitism**. Although, the parasitism scale can only be applied to mycorrhizal plants, the results for non-mycorrhizal plants were arranged in the same way for reasons of clarity. For calculation of RNE please see Materials and Methods Section. Different letters indicate significant differences at *P* = 0.05 (ANOVA). Means ± s.e., *n* = 5.

Biomass in AM *C. canescens* in the interspecific competition treatment decreased with increasing parasitism potential from 3.82 to 0.29 mg (Supplementary Table S1) as indicated by score distribution on the first and second component of the PLS (Figure 1A). Consequently, plant N and plant P showed the same trend. In the NM treatments, this trend was less pronounced (Figure 1B). X-loadings in the PLS plots showed that AM plants in intraspecific competition exhibited least biomass (Figure 1A), with reductions of ~68%, respectively, 71% compared to plants in interspecific competition and single plants (Supplementary Table S1). Hence, AM plants in intraspecific competition had significantly higher RNE (0.52–0.88; *p* < 0.05) than plants in interspecific competition (−0.19 to 0.68), except from shaded plants with high parasitism potential, where RNE values were similar in both intra- and interspecific competition treatments (Figure 2A). Competition and single treatments were significant predictors for biomass (Table 1). Biomass, plant P, and plant N in NM *C. canescens* were highest in interspecific competition (~5.06 g, ~11.84 mg, ~91.78 mg) and lowest in intraspecific competition (~0.81 g, ~1.51 mg, ~11.13 mg; Supplementary Table S1), indicated by opposite X-loadings (Figure 1B). Component 1 explained 48.1% of Y-variance (Table 2). Both interspecific and intraspecific competition were significant predictors for biomass, plant P and plant N (Table 1). Similar to the AM plants, NM plants in intraspecific competition had significantly higher RNE (0.70–0.92; *p* < 0.05) than plants in interspecific competition (−0.26 to 0.49; Figure 2B).

MGD was lowest in the interspecific competition treatment with ~−67% and highest in the single treatment and the intraspecific competition treatment with ~−59 and ~−50%, respectively (Figure 1A, Supplementary Table S1). In addition, MGD in the interspecific competition treatment decreased with increasing parasitism potential (Supplementary Table S1). As indicated by the negative MGD values, AM *C. canescens* in competition was smaller (~0.52 g in intraspecific competition, ~1.63 g in interspecific competition) than in the NM-state (~0.81 g in intraspecific competition, ~5.06 g in interspecific competition; Supplementary Table S1), which was significant (*p* < 0.05) for almost all plants in interspecific competition.

Conditions promoting parasitism did not affect the outcome of competition in *H. pilosella*. As indicated by PLS loadings on component 1 (Figure 1C), AM single plants exhibited highest biomass with ~0.42 mg in contrast to plants in interspecific competition which showed lowest biomass with ~0.13 mg (Supplementary Table S1). Moreover, the single and interspecific competition treatments were significant predictors for biomass (Table 1). Similar to biomass, both plant P and plant N were highest in single plants and lowest in interspecific competition, as indicated by opposite X-loadings on components 1 and 2 (Figure 1C). Correspondingly, RNE in plants grown in interspecific competition was higher (0.48–0.82) than in intraspecific competition (−0.12 to 0.60; Figure 2A). In NM *H. pilosella*, the same trends showed with highest total dry weight (~0.08 g) and plant N (~0.71 mg) in single plants and lowest total dry weight (~0.009 g) and plant N (~0.12 mg) in interspecific competition (Figure 1D; Supplementary Table S1). RNE was higher in interspecific competition (−0.08 to 0.95) than in intraspecific competition (−0.36 to 0.82; Figure 2B). However, these trends were less pronounced than in AM *H. pilosella*. Accordingly, competition treatments were no significant predictors for biomass and plant N in NM *H. pilosella* (Table 1). MGD was lowest in single plants with ~78% and highest in intraspecific and interspecific competition with ~88 and ~92%, respectively (Figure 1C, Supplementary Table S1). In addition, interspecific competition and single treatments were significant predictors for MGD (Table 1). As indicated by MGD values, almost all NM *H. pilosella* plants were significantly smaller than AM *H. pilosella* plants (*p* < 0.05), except from NM single plants at light (~0.14 g) and no fertilization (~0.15 g), which did not differ significantly from AM plants in intraspecific (~0.31 g) and interspecific (~0.13 g) competition treatments (Supplementary Table S1). NM *H. pilosella* plants always exhibited less biomass (~0.04 g) than NM *C. canescens* (~3.46 g), which was significant (*p* < 0.01) for all treatments. Overall, interspecific competition was stronger in *H. pilosella* than in *C. canescens* with RNE values of ~0.68 and ~0.21 for AM plants and ~0.55 and ~0.04 for NM plants (Figure 2). Furthermore, AM *H. pilosella* plants in interspecific competition were smaller (~0.13 g) than the corresponding *C. canescens* plants (~1.63 g, *p* < 0.05), which was significant for all treatments, except for shaded *C. canescens* (~0.83 g; Supplementary Table S1).

In addition to the highly significant main effects of all four factors (species, mycorrhization, competition, and parasitism scale) on biomass (*p* < 0.001; Table 3), significant interactions were found for all factor combinations (*p* < 0.05), except for “species × mycorrhization × competition,” “species × competition × parasitism scale,” “mycorrhiza × competition × parasitism scale,” and the combination of all four factors (Table 3).

**Table 3:** **Summary of four-factorial ANOVA (Fisher's LSD ***post-hoc*** test) on biomass data, with factors species (sp), mycorrhiza (myc), competition (comp), and parasitism scale (para)**.

**Effect**	**Sum of squares**	**Degree of freedom**	**Mean squares**	***F***	***p***
Intercept	249.49	1	249.49	40605.04	<**0.001**
Species (sp)	9.13	1	9.13	1485.87	<**0.001**
Mycorrhiza (myc)	0.33	1	0.33	53.18	<**0.001**
Competition (comp)	0.71	2	0.35	57.69	<**0.001**
Parasitism scale (para)	0.45	5	0.09	14.50	<**0.001**
Sp × myc	2.78	1	2.78	452.41	<**0.001**
Sp × comp	0.83	2	0.41	67.29	<**0.001**
Sp × para	0.33	5	0.07	10.70	<**0.001**
Myc × comp	0.07	2	0.03	5.44	<**0.01**
Myc × para	0.08	5	0.02	2.59	<**0.05**
Comp × para	0.16	10	0.02	2.55	<**0.01**
Sp × myc × comp	0.03	2	0.02	2.78	0.064
Sp × myc × para	0.07	5	0.01	2.30	<**0.05**
Sp × comp × para	0.07	10	0.01	1.07	0.383
Myc × comp × para	0.09	10	0.01	1.42	0.171
Sp × myc × comp × para	0.08	10	0.01	1.24	0.263
Error	1.61	262	0.01		

Significant effects are printed bold (p < 0.05).

## Discussion

### The occurrence of AMF parasitism

AMF parasitism occurs if mycorrhizal C costs exceed nutritional benefits (Johnson et al., 1997). Although up to 20% of photosynthetically derived C can be allocated to the AMF (Jakobsen and Rosendahl, 1990), it has been argued that these costs may be negligible to the plant (Smith et al., 2009; Johnson et al., 2015). Nevertheless, C limitation is still expected to change the threshold for mycorrhizal costs exceeding benefits (Johnson et al., 2015). If soil P is not limiting, AMF are less likely to provide growth benefits (Johnson, 2010). In consequence, the cost:benefit ratio of the symbiosis may become unbalanced and the relationship can turn into parasitism (Johnson et al., 1997), resulting in negative mycorrhizal growth responses (e.g., Smith and Smith, 2013). Several studies have shown that this is most likely in P-rich soils with limited light (e.g., Grman, 2012; Johnson et al., 2015). Equally important for the outcome of the symbiosis is the amount of N in the soil (e.g., Johnson et al., 2003), with N-supply being a driver of photosynthetic capacity (Ellsworth and Reich, 1993) and thus C supply to plant and AMF. Even though N is not the main nutrient delivered by AMF (e.g., Smith and Read, 2008), N:P ratio of the soil is a key determinant for mycorrhizal functioning with the potential for mycorrhizal parasitism decreasing with increasing N:P ratio (Johnson et al., 2003; Johnson, 2010).

In this experiment and in contrast to our first hypothesis, AMF parasitism did not occur in a gradient caused by particular environmental or edaphic conditions but MGD was rather species-specific. As opposed to our second hypothesis that the AMF has no influence on the growth of *C. canescens*, this species showed a markedly negative MGD throughout all treatments, thus indicating consistent AMF parasitism in this species. In a previous pot study, mycorrhizal responsiveness of *C. canescens* to *R. irregularis* has been described as neutral (Höpfner et al., 2014) and *C. canescens* is generally considered facultatively mycotrophic (Wang and Qiu, 2006; Hempel et al., 2013) with quite variable colonization. Negative MGDs in *C. canescens* in this study may be explained by the fact that, in contrast to pot experiments (e.g., Höpfner et al., 2014), there was no spatial limitation for root growth in our experimental setup, leading to better nutrient availability for NM plants and thus reduced mycorrhizal benefits. However, the negative MGDs observed were not clearly related to conditions promoting mycorrhizal parasitism, as although shade and P fertilization had strong negative effects on the growth of AM *C. canescens*, similar effects were found for NM *C. canescens*. These negative effects of shade and P fertilization on growth of NM *C. canescens* may be explained by limited C assimilation and a mild form of P-toxicity (e.g., Silber et al., 2002), respectively. Another reason could be a strengthening of N limitation through P fertilization (Mulder et al., 2013). As biomass N: P ratios are a key factor determining plant growth, a lower N: P ratio in the P addition treatments may have led to lower biomass production in NM *C. canescens* (Güsewell, 2004). This concept may also apply for soil microorganisms other than mycorrhiza, which may pose potential competitors for soil nutrients (Zhu et al., 2016), with P fertilization increasing competition for N in both AM and NM treatments.

Nevertheless, although growth depressions with shade and P fertilization as conditions promoting parasitism occurred in both AM and NM *C. canescens*, both factors were negatively related to MGD (Figure 1A), which, however, was only pronounced in the intra- and interspecific competition treatments. Thus, under competition, AMF had the most negative influence on the growth of *C. canescens* under conditions in which detrimental effects of the symbiosis were presumed, supporting our first hypothesis. This is an indication of mycorrhizal cost:benefit ratio in *C. canescens* being increased by parasitism promoting conditions. However, root colonization in this species was generally very low. In consequence, excessive C drain by the AMF seems to be an unlikely explanation for the negative growth responses (Smith et al., 2010; Smith and Smith, 2012; Lekberg and Koide, 2014). Nonetheless, several studies showed that large growth depressions in AM plants are not necessarily related to high colonization (e.g., Grace et al., 2009; Smith et al., 2010). Instead, it has been argued that P deficiency in these plants may also be caused by a suppression of the root uptake pathway for P in favor of the under low colonization relatively inefficient fungal pathway (Smith et al., 2009). Even though this was questioned by some authors (Lekberg and Koide, 2014), our data lend support to this theory with *C. canescens* exhibiting consistently lower tissue P in the AM than in the NM state (Supplementary Table S1). A second explanation for the observed growth depressions in AM *C. canescens* may be direct competition for N with the AMF, as the fungus itself may pose a strong sink for N and thus rarely improves plant N nutrition in N-deficient soils (Johnson, 2010). Indeed, *C. canescens* showed consistently lower N content in the AM than in the NM state (Supplementary Table S1). N addition alleviated fungal N competition, which resulted in a positive correlation of N fertilization with MGD (Figure 1A).

Overall, and in contrast to our first hypothesis, we found that the mycorrhizal parasitism observed in *C. canescens* was not mainly caused by increased fungal C costs or decreased plant P benefits with shade and P fertilization, factors which have been identified to be of highest importance for a shift in cost:benefit ration of the symbiosis (Johnson et al., 1997). According to our results, negative MGDs in *C. canescens* were rather a result of generally negative effects of mycorrhization in this species and most likely fungal N competition in the nitrogen deficient substrate. This is supported by the negative correlation between root colonization and MGD, indicating that the degree of colonization was driving negative mycorrhizal effects on *C. canescens*. In accordance, Unger et al. (2016) found hyphal density and the degree of colonization governing N-limitation in the studied species.

In marked contrast to *C. canescens*, and in contrast to our second hypothesis, conditions promoting parasitism did not have a noticeable impact on the growth of AM *H. pilosella*. Although colonization was very high in all treatments, there was no pronounced effect of shade and P fertilization on productivity of *H. pilosella* when mycorrhizal (Figure 1C). *H. pilosella*, being an obligate mycotrophic species without the ability to sustain growth and reproduction without an AMF symbiont (Höpfner et al., 2014, 2015), revealed distinctly positive MGDs in all treatments and significantly higher amounts of tissue P in the AM than in the NM state. Nevertheless, treatments affected P-acquisition, with plant P in AM *H. pilosella* being highest in light and lowest in shade (Supplementary Table S1). This is in line with Olsson et al. (2010), who found that shade rather than P fertilization may have a parasitic effect on the symbiotic interaction. Plants supplied C to the AMF even when shaded, which could be considered parasitical behavior of the AMF (Olsson et al., 2010). However, as stated above, lower P-contents of shaded AM *H. pilosella* did not affect biomass. On the contrary, the mycorrhizal growth response was even higher in shaded plants than in the light treatment (Supplementary Table S1), indicating that C limitation due to higher cost:benefit ratios in the shade was not an issue for *H. pilosella*. In many other studies, shading either reduced plant growth and MGD (e.g., Olsson et al., 2010; Konvalinková et al., 2015; Zheng et al., 2015) or did not lead to significant biomass differences between shaded and non-shaded plants (Knegt et al., 2016). However, Johnson et al. (2015) found an increased mycorrhizal growth response at a 33% reduction of light and assumed that the C costs of the AMF in that case may be essentially lower than the plant's photosynthetic capacity. Similar to *C. canescens*, N fertilization correlated positively with MGD (Figure 1C) suggesting N limitation and hence possible competition for N with the AMF (Johnson, 2010).

### Control in the symbiosis and implications for competition

Dominance of fungal over plant control in mycorrhizal symbioses has been widely debated in recent years (e.g., Kiers and van der Heijden, 2006; Kiers et al., 2011; Smith and Smith, 2011; Fellbaum et al., 2014). AMF being obligate biotrophs and hence, dependent on plant C supply, may suggest that the symbiotic interaction is mainly controlled by the plant. For example, van der Heijden and Horton (2009) state that many plants can, at least partly, control colonization under less beneficial conditions such as high P availability. Similarly, Kiers et al. (2011) found plant C investment to depend on cooperativeness in P-delivery of different fungal symbionts. They concluded that a generally reciprocal exchange of nutrients stabilizes the symbiosis. However, many studies have shown contrasting results (Fitter, 2006; Smith and Smith, 2011; Fellbaum et al., 2014), demonstrating that plant dominance of the symbiosis is a simplified view. For instance, Treseder and Allen (2002) found that AMF biomass did not decrease in fertilized soil, indicating that plants did not control the symbiosis under decreased mycorrhizal benefits. Further, experiments with different AMF species showed that plants did not replace less beneficial AMF with more beneficial ones because they either are physically unable to do so or benefit otherwise from these AMF (Verbruggen et al., 2012). Additionally, AMF may considerably control the symbiosis in case of “hidden P uptake,” i.e., the suppression of the root uptake pathway in favor of the fungal pathway (Smith and Smith, 2011). Our results suggest that neither *C. canescens* nor *H. pilosella* controlled their symbiont under conditions with supposedly decreased mycorrhizal benefits. This lent support to our third hypothesis only in case of *H. pilosella*, while we expected *C. canescens* to control mycorrhization. However, in case of *C. canescens*, colonization was not lowered even though AMF induced considerable disadvantages, at least measured in growth response. However, it is possible that AMF still would increase plant fitness in the long term, for instance by improving stress tolerance or fecundity (Jones and Smith, 2004; Smith et al., 2010). As root colonization in the highly mycotrophic *H. pilosella* was neither related to MGD nor changed with conditions promoting parasitism, symbiotic control by AMF is also probable in this species. However, as mycorrhizal benefits in *H. pilosella* were high under all circumstances, there was likely no necessity to downregulate colonization.

AMF have been described to considerably affect competitive interactions (e.g., van der Heijden et al., 1998; Scheublin et al., 2007; Smith et al., 2010; Wagg et al., 2011), for example by mediating competition via common mycelial networks (e.g., Facelli et al., 2010). In this study, both plant species competed mainly for N, since they were generally N-limited (tissue N:P ratio < 14; Koerselman and Meuleman, 1996), except for *H. pilosella* in interspecific competition with obvious P-limitation (N:P ratio >16) in the N fertilization treatments (Supplementary Table S1). The impact of AMF on competition for soil nutrients in both species was modulated by colonization and environmental conditions. The lowest MGD in *C. canescens* was observed in combination with significantly higher root colonization in the interspecific competition treatments. Furthermore, conditions promoting parasitism had detrimental effects on the growth of *C. canescens* when grown with the highly mycotrophic competitor *H. pilosella*. Thus, the high root colonization of *H. pilosella* probably led to markedly higher colonization of *C. canescens* in interspecific competition and concomitantly lower MGD with increasing parasitism potential, which was not observed in the intraspecific competition and single treatments (Figure 1A). Competitive effects as driven by mycorrhization in both species were most pronounced in the interspecific competition treatments, however, for different reasons. Negative mycorrhizal growth responses of *C. canescens* were possibly amplified by higher colonization in presence of *H. pilosella*. These effects were most detrimental under conditions promoting parasitism, which was also underlined by RNE values (Figure 2A). They indicated that *C. canescens* actually experienced stronger competition from other *C. canescens* plants than from *H. pilosella*, except for shaded plants, where RNE values of interspecific competition treatments were similarly high as in intraspecific competition. Thus, the outcome of competition did not change depending on whether the AMF operated mutualistically or parasitically, but enhanced the existing deleterious effects of mycorrhization on *C. canescens*. Contrastingly, root colonization in *H. pilosella* was reduced in presence of *C. canescens* (Figure 1C), leading to markedly decreased biomass, plant N and plant P as compared to single plants. Lowered root colonization in *H. pilosella* in presence of *C. canescens* also resulted in severe P-limitation. Nonetheless, even in competition with *C. canescens* when mycorrhization decreased, AM *H. pilosella* still exhibited consistently larger biomass than NM plants. This could possibly change in a more natural system with two or more AMF species being differently beneficial (e.g., Dodd et al., 2000; van der Heijden et al., 2003; Egger and Hibbett, 2004). Therefore, the use of other AMF-species in the present experimental setup is desirable to be considered in future studies, as AMF-identity may yield differential effects on growth response and in consequence on the mediation of competitive interactions (e.g., van der Heijden et al., 1998; Klironomos, 2003).

It has been suggested that less mycotrophic plants, potentially investing little resources in the symbiosis, will have a competitive advantage under conditions promoting parasitism (Johnson et al., 2015). Indeed, in the mycorrhizal state, *H. pilosella* clearly was the weaker competitor (Figure 2A), in general exhibiting considerably larger RNE than *C. canescens* in interspecific competition. However, a competitive disadvantage of *H. pilosella* with conditions promoting mycorrhizal parasitism (see hypothesis 4) was not observed, while this was true for *C. canescens*. In contrast to these results, Zabinski et al. (2002) stated that a more mycotrophic plant would easily exploit the neighbor's rooting zone with extraradical hyphae and thus would have advantages over a less mycotrophic competitor. In addition, Wilson et al. (2006) found that the majority of mycorrhizal P transfer in common mycelial networks was directed to the more responsive species. On the other hand, Höpfner et al. (2015) showed AM *H. pilosella* to suffer from competition with *Plantago lanceolata*, a less mycotrophic species pursuing a more root-dominated foraging strategy. This indicates that AMF-dominated foraging can be less effective than root-dominated foraging in competitive interactions, particularly under high nutrient availabilities. Our results indicate that host choice and control of colonization, and moreover control of competitive interactions, by the AMF rather than by the plant, may determine the outcome of competition for soil nutrients. This is in line with results of Fellbaum et al. (2012), describing that AMF can be in control of the symbiosis and moreover, can discriminate against hosts which supply less C. In our experiment, increased colonization of *C. canescens* and decreased colonization of *H. pilosella* indicates that in the interspecific competition treatment, the AMF possibly had greater advantages from the symbiosis with *C. canescens* than with *H. pilosella*, despite fairly low colonization levels. One possible reason is the greater amount of C that *C. canescens* can supply with its considerably larger leaf biomass, i.e., Fellbaum et al. (2014) showed that AMF allocated relatively more nutrients to individual hosts with highest carbon source strength in a common mycelial network. Modulating colonization of interlinked hosts may further be a mechanism of AMF to mitigate competition and increase the survival of potential hosts, as has been observed in other studies, where seedlings received increased AMF benefits if adult neighbor plants were defoliated (Pietikäinen and Kytöviita, 2007), with higher colonization of a more secure host being an escape strategy of AMF in case of a loss (Olsson et al., 2010).

### Conclusions

Our results showed that the mutualism-parasitism continuum as established by Johnson et al. (1997) did not apply, with the experimental manipulation of light, N and P having only minor effects on MGD in both species. The high mycotrophy of *H. pilosella* did not allow for the symbiotic outcome to switch into AMF parasitism, as mycorrhizal benefits never vanished in this species. In contrast, MGD in *C. canescens* was generally negative, albeit largely independent from environmental or edaphic conditions. Thus, the hypothesized relationship between the degree of mycotrophy and the extent of detrimental AMF-effects on productivity could not be confirmed. However, AMF was shown to strongly affect interspecific competitive interactions, most likely by modulation of the degree of colonization in both competitors. The lack of control over mycorrhizal colonization thus potentially impaired both species, resulting in lower biomass under interspecific competition. Even though for *C. canescens* environmental conditions potentially inducing mycorrhizal parasitism were shown to decrease MGD while increasing RNE under competition with *H. pilosella*, no clear evidence for a decreased C-cost:P-benefit ratio governing competitive interactions of both species could be found. In contrast, negative effects of mycorrhization were most likely caused by fungal N competition, with this effect being amplified by higher colonization in presence of *H. pilosella*.

Thus, the lack of plant control over mycorrhizal colonization was identified as a possible key factor for the outcome of competition, while environmental and edaphic conditions affecting the mycorrhizal C-cost:P-benefit ratio appeared to be less important. Our findings highlight the need for future field experiments focusing on the extent of symbiotic control by both AMF and host plants and their relation to the outcome of competition in interspecific common mycelial networks.

## Author contributions

MF designed the experiment, accomplished the experimental work, data analysis, and writing. SU assisted with the experimental work, data interpretation, and writing. CH accomplished the statistical analysis and data presentation. WB assisted with data interpretation and writing.

## Funding

We acknowledge support for the Article Processing Charge by the Deutsche Forschungsgemeinschaft and the Open Access Publication Fund of Bielefeld University.

### Conflict of interest statement

The authors declare that the research was conducted in the absence of any commercial or financial relationships that could be construed as a potential conflict of interest.
