# Supplementary material for: Conditions Promoting Mycorrhizal Parasitism Are of Minor Importance for Competitive Interactions in Two Differentially Mycotrophic Species
Source: Front Plant Sci. 2016 Sep 27;7:1465. doi: 10.3389/fpls.2016.01465 (PMC5037182; doi:10.3389/fpls.2016.01465)
Supplement: Supplementary file 1 [file Table1.DOCX]

Supplementary Material

**Competitive Interactions between Two Differentially Mycotrophic Species along the Mycorrhizal Mutualism-Parasitism Continuum**

**Martina Friede*, Stephan Unger, Christine Hellmann, Wolfram Beyschlag**

***Correspondence:** Martina Friede: [friede@uni-bielefeld.de](mailto:friede@uni-bielefeld.de)

**Supplementary Table S1.** Total biomass, plant nitrogen (N) content, plant phosphorus (P) content, mycorrhizal growth dependency (MGD) and root colonization of mycorrhizal (AM) and non-mycorrhizal (NM) *Corynephorus canescens* (CC) and *Hieracium pilosella* (HP) as single plants, in intraspecific and interspecific competition. Treatment numbering from 1 to 5 indicates treatments with low to high mycorrhizal parasitism potential on a theoretical scale. Means and standard errors (s. e.), n=5.

| Treatment | Parameter | | | | | | | | | |
| --- | --- | --- | --- | --- | --- | --- | --- | --- | --- | --- |
|  | Total biomass  [g] | | Plant N [mg] | | Plant P [mg] | | MGD [%] | | Root colonization  [%] | |
|  | Mean | S. e. | Mean | S. e. | Mean | S. e. | Mean | S. e. | Mean | S. e. |
| CC AM Single 1 | 2.3302 | 0.0496 | 33.9313 | 4.3425 | 3.6035 | 0.4664 | -66.85 | 0.71 | 3.7 | 0.7 |
| CC AM Single 2 | 3.1774 | 0.9379 | 43.3321 | 18.6856 | 10.1636 | 4.9938 | -46.33 | 15.79 | 2.8 | 1.1 |
| CC AM Single 3a | 1.9311 | 0.4128 | 31.7570 | 6.6314 | 3.3453 | 1.2221 | -59.26 | 8.33 | 5.8 | 2.3 |
| CC AM Single 3b | 0.8661 | 0.2387 | 8.3793 | 2.7705 | 1.0399 | 0.2879 | -82.17 | 4.91 | 1.4 | 0.9 |
| CC AM Single 4 | 1.8458 | 0.6223 | 18.0966 | 6.2448 | 3.4151 | 1.3298 | -23.69 | 21.79 | 0.8 | 0.4 |
| CC AM Single 5 | 0.5888 | 0.1383 | 5.1587 | 1.3803 | 0.8532 | 0.1650 | -72.45 | 6.47 | 0.2 | 0.2 |
| CC AM Intra 1 | 1.2489 | 0.3011 | 25.9584 | 7.6599 | 2.3782 | 0.7001 | -29.63 | 16.70 | 3.4 | 2.7 |
| CC AM Intra 2 | 0.3904 | 0.1170 | 3.4387 | 0.9590 | 0.5161 | 0.1541 | -22.69 | 19.96 | 5.4 | 3.5 |
| CC AM Intra 3a | 0.5683 | 0.1877 | 11.3612 | 4.0173 | 0.9642 | 0.4712 | -33.88 | 19.83 | 7.2 | 2.4 |
| CC AM Intra 3b | 0.4006 | 0.1508 | 3.0584 | 1.2967 | 0.4800 | 0.2170 | -34.44 | 23.58 | 5.4 | 1.9 |
| CC AM Intra 4 | 0.2337 | 0.0456 | 1.6390 | 0.3540 | 0.2578 | 0.0274 | -59.73 | 7.86 | 5.4 | 3.5 |
| CC AM Intra 5 | 0.3139 | 0.0885 | 3.8613 | 1.6918 | 0.7931 | 0.2917 | -50.60 | 13.92 | 9.8 | 2.8 |
| CC AM Inter 1 | 3.8182 | 0.8732 | 85.6747 | 19.6754 | 10.5869 | 4.9113 | -66.15 | 7.74 | 20.4 | 6.7 |
| CC AM Inter 2 | 2.6686 | 0.0756 | 35.8886 | 4.2952 | 7.5470 | 1.5972 | -11.78 | 2.50 | 17.3 | 7.3 |
| CC AM Inter 3a | 1.6046 | 0.3990 | 39.8802 | 9.2695 | 3.3540 | 1.0115 | -72.13 | 6.93 | 15.4 | 3.6 |
| CC AM Inter 3b | 0.8285 | 0.2013 | 8.1490 | 2.4438 | 1.3143 | 0.4325 | -81.34 | 4.53 | 27.2 | 3.5 |
| CC AM Inter 4 | 0.5933 | 0.1512 | 5.0515 | 1.6525 | 1.1205 | 0.3562 | -80.97 | 4.85 | 20.6 | 0.9 |
| CC AM Inter 5 | 0.2917 | 0.0581 | 2.5822 | 0.5456 | 0.4543 | 0.1087 | -89.44 | 2.10 | 21.8 | 6.2 |
| HP AM Single 1 | 0.4434 | 0.1385 | 6.1757 | 1.8626 | 0.4854 | 0.3034 | 95.78 | 1.27 | 59.4 | 5.2 |
| HP AM Single 2 | 0.6085 | 0.1619 | 5.0826 | 1.2565 | 1.9216 | 0.4963 | 40.95 | 15.32 | 72.8 | 9.2 |
| HP AM Single 3a | 0.5331 | 0.0203 | 8.5564 | 0.6394 | 0.8563 |  | 98.78 | 0.05 | 53.3 | 6.4 |
| HP AM Single 3b | 0.2872 | 0.0347 | 2.8648 | 0.3807 | 0.6500 | 0.0817 | 49.54 | 8.28 | 59.8 | 4.6 |
| HP AM Single 4 | 0.2999 | 0.0603 | 2.0220 | 0.4941 | 0.4052 | 0.3328 | 88.81 | 2.90 | 75.2 | 5.5 |
| HP AM Single 5 | 0.3545 | 0.0852 | 2.7206 | 0.9345 | 0.6447 | 0.2617 | 96.58 | 0.79 | 53.0 | 12.4 |
| HP AM Intra 1 | 0.6224 | 0.1726 | 10.3085 | 3.3721 | 0.8257 | 0.5989 | 95.51 | 0.94 | 66.4 | 6.4 |
| HP AM Intra 2 | 0.2452 | 0.0585 | 2.2901 | 0.4537 | 0.0000 |  | 72.09 | 8.54 | 71.6 | 5.3 |
| HP AM Intra 3a | 0.3067 | 0.0683 | 6.3650 | 1.2058 | 0.2238 | 0.0421 | 92.97 | 2.13 | 71.4 | 1.8 |
| HP AM Intra 3b | 0.1668 | 0.0253 | 1.3391 | 0.1923 | 0.6483 | 0.0779 | 82.25 | 2.26 | 71.0 | 5.2 |
| HP AM Intra 4 | 0.2649 | 0.0324 | 1.6597 | 0.2118 | 0.2729 | 0.0376 | 95.55 | 0.68 | 76.0 | 5.6 |
| HP AM Intra 5 | 0.2272 | 0.0719 | 1.4552 | 0.4341 | 0.4167 | 0.1549 | 89.09 | 3.06 | 67.6 | 5.2 |
| HP AM Inter 1 | 0.0882 | 0.0157 | 1.4974 | 0.2797 | 0.0457 | 0.0205 | 91.58 | 1.59 | 56.8 | 2.7 |
| HP AM Inter 2 | 0.2320 | 0.0330 | 1.7683 | 0.2089 | 0.3370 | 0.0330 | 93.43 | 0.76 | 46.6 | 8.2 |
| HP AM Inter 3a | 0.0961 | 0.0196 | 1.8433 | 0.3176 | 0.0277 | 0.0158 | 89.95 | 2.71 | 54.3 | 3.0 |
| HP AM Inter 3b | 0.0863 | 0.0101 | 0.6142 | 0.1442 | 0.1858 | 0.0377 | 84.94 | 1.61 | 49.0 | 3.4 |
| HP AM Inter 4 | 0.1570 | 0.0156 | 0.7963 | 0.0894 | 0.1202 | 0.0313 | 95.04 | 0.51 | 35.0 | 4.9 |
| HP AM Inter 5 | 0.1229 | 0.0160 | 0.7187 | 0.0887 | 0.1630 | 0.0340 | 94.75 | 0.60 | 36.2 | 6.7 |
| CC NM Single 1 | 7.0291 | 1.2108 | 99.6872 | 22.2175 | 16.1838 | 4.2784 |  |  | 0 | 0 |
| CC NM Single 2 | 5.9085 | 1.6688 | 74.2200 | 27.5584 | 16.0049 | 6.3848 |  |  | 0 | 0 |
| CC NM Single 3a | 4.9579 | 0.4705 | 76.2750 | 15.9384 | 10.6161 | 1.9458 |  |  | 0 | 0 |
| CC NM Single 3b | 4.8579 | 1.3469 | 54.9099 | 22.5501 | 11.3487 | 3.8174 |  |  | 0 | 0 |
| CC NM Single 4 | 2.1963 | 0.3625 | 21.1481 | 5.2219 | 3.7701 | 1.0066 |  |  | 0 | 0 |
| CC NM Single 5 | 2.1372 | 0.6521 | 22.5875 | 5.3523 | 3.3578 | 0.8576 |  |  | 0 | 0 |
| CC NM Intra 1 | 1.7589 | 0.4220 | 31.0179 | 8.1402 | 4.0368 | 0.9944 |  |  | 0 | 0 |
| CC NM Intra 2 | 0.4693 | 0.0815 | 5.0038 | 2.0722 | 0.8299 | 0.2267 |  |  | 0 | 0 |
| CC NM Intra 3a | 0.8146 | 0.3040 | 17.9880 | 7.3098 | 1.5368 | 0.6287 |  |  | 0 | 0 |
| CC NM Intra 3b | 0.5807 | 0.1422 | 4.5011 | 2.0908 | 0.8926 | 0.2364 |  |  | 0 | 0 |
| CC NM Intra 4 | 0.5803 | 0.0221 | 3.6223 | 0.2040 | 0.7355 | 0.0929 |  |  | 0 | 0 |
| CC NM Intra 5 | 0.6354 | 0.1913 | 4.6255 | 1.3076 | 1.0131 | 0.2991 |  |  | 0 | 0 |
| CC NM Inter 1 | 11.2813 | 2.2340 | 248.1709 | 47.7209 | 31.2872 | 7.7297 |  |  | 0 | 0 |
| CC NM Inter 2 | 3.0251 | 1.3188 | 40.7157 | 24.1038 | 5.9445 | 4.0641 |  |  | 0 | 0 |
| CC NM Inter 3a | 5.7575 | 1.3734 | 129.9615 | 33.4537 | 12.3094 | 3.0805 |  |  | 0 | 0 |
| CC NM Inter 3b | 4.4402 | 1.6046 | 59.6675 | 28.2932 | 10.1885 | 4.4350 |  |  | 0 | 0 |
| CC NM Inter 4 | 3.1178 | 0.6405 | 32.4535 | 7.9120 | 5.6903 | 1.4849 |  |  | 0 | 0 |
| CC NM Inter 5 | 2.7616 | 0.9641 | 39.7029 | 21.3336 | 5.6434 | 2.6499 |  |  | 0 | 0 |
| HP NM Single 1 | 0.0121 | 0.0053 | 0.1601 | 0.0775 | 0.0194 |  |  |  | 0 | 0 |
| HP NM Single 2 | 0.2684 | 0.1170 | 2.7111 | 1.1220 | 0.7623 | 0.2253 |  |  | 0 | 0 |
| HP NM Single 3a | 0.0065 | 0.0020 | 0.0828 | 0.0333 |  |  |  |  | 0 | 0 |
| HP NM Single 3b | 0.1337 | 0.1004 | 0.9838 | 0.7219 | 1.1186 |  |  |  | 0 | 0 |
| HP NM Single 4 | 0.0272 | 0.0137 | 0.2162 | 0.1023 |  |  |  |  | 0 | 0 |
| HP NM Single 5 | 0.0095 | 0.0034 | 0.1070 | 0.0402 | 0.0042 |  |  |  | 0 | 0 |
| HP NM Intra 1 | 0.0220 | 0.0077 | 0.5205 | 0.3788 | 0.0112 |  |  |  | 0 | 0 |
| HP NM Intra 2 | 0.0490 | 0.0439 | 0.4558 | 0.3695 |  |  |  |  | 0 | 0 |
| HP NM Intra 3a | 0.0163 | 0.0064 | 0.2545 | 0.1582 | 0.0023 |  |  |  | 0 | 0 |
| HP NM Intra 3b | 0.0274 | 0.0115 | 0.3002 | 0.2664 |  |  |  |  | 0 | 0 |
| HP NM Intra 4 | 0.0109 | 0.0011 | 0.4558 | 0.3695 |  |  |  |  | 0 | 0 |
| HP NM Intra 5 | 0.0175 | 0.0036 | 0.1888 | 0.0420 |  |  |  |  | 0 | 0 |
| HP NM Inter 1 | 0.0067 | 0.0020 | 0.1832 | 0.0110 | 0.0011 | 0.0011 |  |  | 0 | 0 |
| HP NM Inter 2 | 0.0143 | 0.0041 | 0.1382 | 0.0312 |  |  |  |  | 0 | 0 |
| HP NM Inter 3a | 0.0081 | 0.0019 | 0.1396 | 0.0442 |  |  |  |  | 0 | 0 |
| HP NM Inter 3b | 0.0125 | 0.0039 | 0.1092 | 0.0303 | 0.0000 |  |  |  | 0 | 0 |
| HP NM Inter 4 | 0.0075 | 0.0024 | 0.1289 | 0.0349 | 0.0007 |  |  |  | 0 | 0 |
| HP NM Inter 5 | 0.0061 | 0.0013 | 0.0696 | 0.0184 |  |  |  |  | 0 | 0 |
